# Persistent socioeconomic and ethnic inequalities in unmet need for NCD diagnosis and treatment in Viet Nam, 2015–2021

**DOI:** 10.1371/journal.pgph.0006952

**Published:** 2026-08-07

**Authors:** Lan Thi Hoang Vu, Quyen Thi Tu Bui, Quy Xuan Luu, Tuan Kim Duong, Minh Van Hoang

**Affiliations:** 1 Faculty of Fundamental Sciences, Hanoi University of Public Health. No. 1A Duc Thang, Dong Ngac Ward, Ha Noi, Viet Nam; 2 Health Management Training Institute, Hanoi University of Public Health. No. 1A Duc Thang, Dong Ngac Ward, Ha Noi, Viet Nam; Directorate of Factories, INDIA

## Abstract

Large gaps in the detection and treatment of noncommunicable diseases (NCDs) persist globally, particularly in low- and middle-income countries progressing toward universal health coverage. Despite expanded insurance and primary care reforms, the magnitude and equity of unmet NCD care needs in Viet Nam remain unclear. We analyzed nationally representative WHO STEPS surveys in 2015 (n = 1,300) and 2021 (n = 2,276) to assess unmet need for diagnosis and treatment of hypertension, diabetes, and dyslipidemia and to examine inequalities by socioeconomic status, education, and ethnicity. Unmet need for diagnosis and unmet need for treatment were analyzed as separate primary outcomes. Inequalities were quantified using the Erreygers Concentration Index (ECI), while absolute and relative inequalities were assessed using the Slope Index of Inequality (SII) and Relative Index of Inequality (RII), with survey weights applied to prevalence and inequality estimates. Unmet need for diagnosis remained high, increasing from 82.2% in 2015 to 84.5% in 2021, while unmet need for treatment was higher but changed little, from 91.9% to 90.9%. Dyslipidemia showed the largest gaps. Ethnic inequalities persisted across survey years, with RII values ranged from 1.25 (95% CI: 1.14-1.36) to 1.29 (95% CI: 1.14-1.46) for unmet diagnosis and from 1.12 (95% CI: 1.03-1.22) to 1.17 (95% CI: 1.10-1.24) for unmet treatment. Socioeconomic inequalities in diagnosis also persisted, with RII values of 0.80 (95% CI: 0.73-0.89) in 2015 and 0.90 (95% CI: 0.84-0.96) in 2021, whereas education-related inequalities observed in 2015 were no longer apparent in 2021. All three inequalities measures showed consistent patterns. Viet Nam continues to face substantial unmet needs for NCD diagnosis and treatment, with persistent ethnic and socioeconomic disparities. Equity-oriented primary care and NCD services are needed to support more equitable progress toward universal health coverage.

## Introduction

Noncommunicable diseases (NCDs), particularly hypertension (HTN), diabetes (DM), and dyslipidemia, are major contributors to premature mortality and disability, accounting for nearly three-quarters of global deaths [1]. In many low- and middle-income countries (LMICs), health systems remain more oriented toward acute care than toward long-term management, leaving the prevention and control of chronic diseases under-resourced [2,3]. Viet Nam, a rapidly developing Southeast Asian country, has experienced a significant increase in NCD prevalence over the past two decades [4]. Although national efforts have expanded health insurance coverage and strengthened primary care, substantial gaps persist in early detection and treatment, threatening progress toward Universal Health Coverage (UHC) and Sustainable Development Goal (SDG) 3.4 on reducing premature NCD mortality [5].

Early diagnosis and sustained treatment are critical to minimizing complications, yet evidence from Southeast Asia shows that large proportions of adults with HTN or DM remain undiagnosed, and even fewer receive appropriate therapy [6–9]. In Viet Nam, previous studies have largely focused on single diseases or clinical control rates, with limited attention to how unmet needs differ across social groups or whether inequalities have changed over time, particularly as the country reforms its health financing and service delivery systems. To better understand these disparities, this study draws on Andersen’s Behavioral Model of Health Services Use [10], which posits that service utilization is influenced by predisposing, enabling, and need factors. Education and ethnicity represent predisposing characteristics that shape awareness, perceptions of illness, and help-seeking behavior, while household wealth functions as an enabling resource that affects affordability and geographic access. Within this framework, the unmet need for diagnosis reflects barriers to initial access, whereas the unmet need for treatment reflects challenges in continuity of care, including medication costs and follow-up appointments.

Despite increasing policy focus on universal health coverage (UHC) [11], little is known about whether inequalities in NCD care are more pronounced at the point of disease detection or during treatment, and whether these disparities have shifted over time. To address this gap, this study aims to (1) estimate and compare the prevalence of unmet need for diagnosis and treatment of HTN, DM, and dyslipidemia among Vietnamese adults using nationally representative STEPS surveys from 2015 and 2021 and (2) assess socioeconomic and ethnic inequalities in these unmet needs using established absolute and relative inequality measures (ECI, SII, and RII).

## Methods

### Ethical statement

This study used anonymized secondary data from the 2015 and 2021 WHO STEPS surveys in Viet Nam. Ethical approval for primary data collection was obtained from the Ministry of Health of Viet Nam and the Hanoi University of Public Health (No. 395/2020/YTCC-HD3 on 25 Sep 2020). All participants provided written informed consent before participation. As the present analysis used de-identified data, no additional institutional review board approval was required.

### Data sources and study population

This study employed a repeated cross-sectional design using nationally representative data from the 2015 and 2021 WHO STEPwise Approach to NCD Risk Factor surveys conducted in Viet Nam. Both surveys followed standardized WHO protocols using a two-stage systematic random sampling design to ensure representativeness across all 63 provinces and cities. While the STEPS surveys target individuals aged 15 years and older, anthropometric and biochemical measurements (STEPS 2 and 3) were restricted to participants aged 18 and above. Therefore, our analytical sample was limited to adults aged ≥18 years with complete biochemical and anthropometric data.

The total number of participants completing Step 3 was 3,074 in 2015 and 3,712 in 2021. For the current analysis, we apply specific selection criteria to identify the study population, which was restricted to individuals with at least one of the following three conditions: (i) Hypertension: defined as systolic blood pressure ≥140 mmHg, diastolic blood pressure ≥90 mmHg, or current use of antihypertensive medication (n = 688 in 2015; n = 1,316 in 2021); (ii) Diabetes: to ensure diagnostic accuracy, the analysis included only participants who fulfilled the fasting requirements of at least 8 hours prior to the biochemical test. DM was defined as fasting blood glucose ≥7.0 mmol/L, or current use of insulin or other glucose-lowering medication (n = 132 in 2015; n = 305 in 2021); and (iii) Dyslipidemia: defined as total cholesterol ≥5.2 mmol/L, or current use of lipid-lowering medication (n = 859 in 2015; n = 1,558 in 2021).

Overall, 1,300 participants in 2015 and 2,276 participants in 2021 met the inclusion criteria of having at least one of the three measured conditions and were included in the final analysis of unmet needs for diagnosis and treatment. The reduction from all Step 3 participants to the final analytical sample reflected disease-specific eligibility criteria rather than missing data. Participants without hypertension, diabetes, or dyslipidemia were not eligible for the unmet-need analyses and were therefore excluded by design.

It is important to note that field data collection for the 2021 survey was completed before the major waves of the COVID-19 pandemic and the subsequent widespread lockdowns in Viet Nam.

### Study measurements

#### Outcomes.

This study examined six disease-specific outcomes and two combined indicators of unmet need.

Hypertension: Unmet need for early diagnosis was defined as the proportion of individuals with measured HTN who had never been told by a health professional that they had high blood pressure. Unmet need for treatment was defined as the proportion of individuals with HTN who were not currently taking antihypertensive medication.

Diabetes: The unmet need for early diagnosis was defined as the proportion of individuals with DM who had never been informed by a healthcare professional that they had the condition. Unmet need for treatment was defined as the proportion of individuals with DM who were not currently taking insulin or other glucose-lowering medications.

Dyslipidemia: Unmet need for early diagnosis was defined as the proportion of individuals with dyslipidemia who had never been informed by a health professional of their condition. Unmet need for treatment was defined as the proportion of individuals not currently taking lipid-lowering medication.

Combined Indicators: Unmet need for early diagnosis was defined among individuals with at least one of the three conditions (HTN, DM, or dyslipidemia) who had never been diagnosed with that condition. Unmet treatment need was defined as the presence of any of the three conditions among individuals who were not currently taking the prescribed medication.

### Independent variables

Sociodemographic characteristics included age group (1824, 25–34, 35–44, 45–54, and ≥55 years), sex (male/female), residence (urban/rural), and ethnicity (Kinh/Hoa or minority group). Education was categorized as primary or less, secondary, high school, university/college, or missing. Socioeconomic status (SES) was measured using a household wealth index derived from principal component analysis (PCA) of household assets and housing characteristics. This method, widely used in national surveys across LMICs, has been validated for use in Viet Nam [12]. The wealth index was divided into quintiles, from the poorest (Q1) to the richest (Q5).

### Statistical analysis

All analyses were conducted using Stata version 19 (StataCorp, College Station, TX, USA). To account for the complex survey design, all analyses incorporated the survey weights, primary sampling units (PSUs), and sampling strata provided in the WHO STEPS datasets. Because the present study was restricted to participants who completed biochemical assessments (Step 3), Step 3 sampling weights (wstep3) were applied throughout the analyses. These weights were developed by the STEPS survey team to account for differential probabilities of selection, non-response, and post-stratification adjustments, thereby ensuring national representativeness.

Survey settings were specified using the Stata command: svyset psu [pweight = wstep3], strata(stratum)

The outcomes of interest were binary variables indicating whether there was an unmet need for diagnosis or treatment. The main determinants of inequality were education, ethnicity, and socioeconomic status (SES). Socioeconomic-related inequality was quantified using the Erreygers-normalized Concentration Index (ECI), which is appropriate for binary outcomes [13]. ECIs were computed for each determinant using the conindex command with the bounded (0, 1) option. Positive ECI values indicate a higher prevalence of unmet need among advantaged groups (pro-rich inequality), while negative values indicate a concentration of unmet need among disadvantaged groups (pro-poor inequality).

To assess both absolute and relative inequalities in unmet needs for NCD diagnosis and treatment, we estimated the Slope Index of Inequality (SII) and the Relative Index of Inequality (RII) using generalized linear models with ridit-transformed determinants (SES and education) [14]. Ridit (Relative to an Identified Distribution) scores represent an individual’s relative position in the cumulative distribution of an ordered social variable, ranging from the most disadvantaged (0) to the most advantaged (1). Categorical socioeconomic variables, SES quintile, education level, and ethnicity, were transformed into ridit scores, representing each participant’s relative position in the cumulative distribution of the determinant. For binary outcomes, SII was derived from linear probability models, which provide the absolute difference in predicted probabilities of unmet need between the top and bottom of the social hierarchy. RII was estimated by Poisson regression models with robust standard errors, which yield the relative ratio of predicted probabilities between the extremes. Separate crude models were fitted for each determinant. As a sensitivity analysis, additional models were adjusted for age group, sex, residence, education, ethnicity, and SES, as appropriate. SII and RII were computed separately for 2015 and 2021. Changes in the magnitude and direction of these indices were interpreted to evaluate whether improvements in NCD care coverage were equitably distributed across social groups. An SII of 0 and an RII of 1 both indicate the absence of socioeconomic inequality.

In this study, positive SII values indicate a higher unmet need for diagnosis or treatment among the more advantaged groups (pro-rich/pro-educated), whereas negative values indicate higher unmet need among the less advantaged groups (pro-poor/pro-less educated). Similarly, RII values greater than 1 represent the fold difference in unmet need probabilities between the most and least privileged individuals, while values less than 1 indicate a fold difference in the opposite direction. Results are reported with 95% confidence intervals, and statistical significance was set at p < 0.05.

Missing data were assessed for all variables included in the analyses. Because the STEPS survey collects questionnaire information (Step 1) and physical measurements (Step 2) before biochemical testing (Step 3), participants who completed Step 3 generally had complete information on demographic and behavioural variables. Missingness was minimal among the analytical sample. Among eligible participants, missingness was low. For sociodemographic variables, only six participants in 2015 had missing information on education; these observations were retained as a separate category (“missing/no information”) and were not excluded from the analyses. For outcome variables, missingness ranged from 0.08% to 6.17%. Specifically, missing diagnosis information ranged from 0.08% to 2.27% across conditions and survey years, while missing treatment information ranged from 0.30% to 6.17%, with the highest level observed for dyslipidemia treatment in 2015 (53/859 participants). S1 Table presents the number and percentage of missing observations for each outcome. Because missingness was low and no variable exceeded 10%, complete-case analysis was considered appropriate and multiple imputations were not performed.

Sensitivity analyses were conducted to assess whether the observed inequality patterns were influenced by differences in sociodemographic composition between the 2015 and 2021 samples. Survey-weighted linear regression models (for SII) and Poisson generalized linear models with a log link (for RII) were additionally adjusted for age group, sex, residence, education, ethnicity, and SES, as appropriate. The adjusted results are presented in S2 Table.

## Results

### Characteristics of the study sample

Table 1 presents the characteristics of adults with at least one chronic condition included in the analysis. Compared with 2015, the 2021 sample included a higher proportion of older adults and those with lower educational attainment. The proportion of urban residents and ethnic minority participants also increased slightly between the two survey years.

**Table 1:** Characteristics of adults with at least one chronic condition in the 2015 and 2021 Viet Nam STEPS surveys.

*Characteristics*	*Year 2015 (n = 1300)*	*Year 2021 (n = 2276)*
Age group		
18-24	39 (3.0%)	48 (2.1%)
25-34	136 (10.5%)	190 (8.3%)
35-44	258 (19.8%)	425 (18.7%)
45-54	392 (30.2%)	593 (26.1%)
over 55	475 (36.5%)	1020 (44.8%)
Education group		
Primary school or less	261 (20.1%)	978 (43.0%)
Secondary school	282 (21.7%)	578 (25.4%)
High school	369 (28.4%)	384 (16.9%)
University/college	382 (29.4%)	336 (14.8%)
No information	6 (0.4%)	
Sex		
Male	564 (43.4%)	1139 (50.0%)
Female	736 (56.6%)	1137 (50.0%)
Location		
Urban	611 (47.0%)	1141 (50.1%)
Rural	689 (53.0%)	1135 (49.9%)
Ethnicity group		
Kinh	1119 (86.1%)	1918 (84.3%)
Others	181 (13.9%)	358 (15.7%)
Household SES		
Q1	253 (19.5%)	426 (18.7%)
Q2	319 (24.5%)	539 (23.7%)
Q3	209 (16.1%)	494 (21.7%)
Q4	272 (20.9%)	417 (18.3%)
Q5	247 (19.0%)	400 (17.6%)

Note: SES, Socioeconomic status; Q, quintiles. Characteristics are reported as unweighted counts and percentages for the analytical sample. All subsequent analyses incorporated survey weights and complex survey design

### Pattern of unmet needs over time

The proportion of unmet need for diagnosis for any of the three diseases was high at 82.2% (95% CI: 79.6 - 84.4) in 2015 and increased slightly to 84.5% (95% CI: 82.3 - 86.5) in 2021. Similarly, the unmet need for treatment for any three diseases remained very high at 91.9% (95% CI: 90.2-93.4) initially, then decreased slightly to 90.9% (95% CI: 89.0-92.6) in the subsequent period.

**Fig 1:**
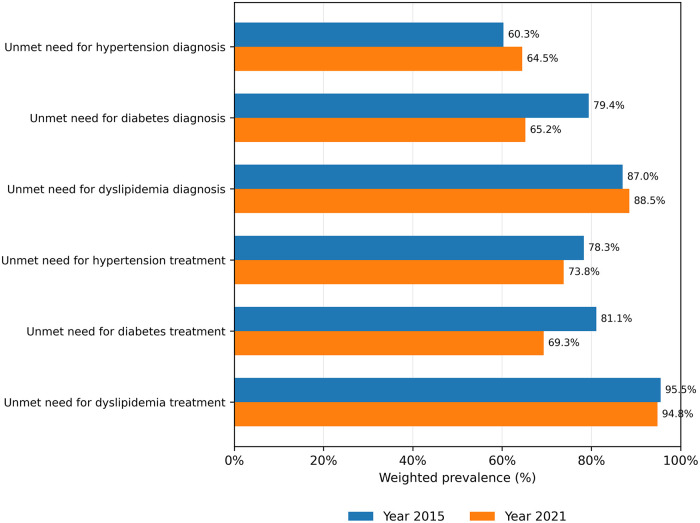
Unmet need for diagnosis and treatment among Vietnamese adults aged 18 years or older with hypertension, diabetes, and dyslipidemia. STEPS 2015 and 2021.

Fig 1 presents the gaps in diagnosis and treatment for each disease. In 2015, the largest diagnosis gaps were observed for dyslipidemia (87.0%), followed by DM (79.4%) and HTN (60.3%). By 2021, unmet needs for diagnosis increased slightly for HTN (64.5%) and decreased for DM (65.2%), while remaining high for dyslipidemia (88.5%). For treatment, dyslipidemia again had the highest unmet needs in both 2015 (95.5%) and 2021 (94.8%), while the unmet need for DM treatment reduced from 81.1% in 2015 to 69.3% in 2021, and HTN showed modest improvements (from 78.3% in 2015 to 73.8% in 2021).

### Concentration Index of Inequality in unmet need for NCDs diagnosis and unmet need for NCDs treatment

In both 2015 and 2021, the overall unmet need for early diagnosis for any of the three diseases was significantly concentrated among disadvantaged populations: the negative ECI for SES (-0.111 and -0.057) indicates that lower socioeconomic groups bore a disproportionate burden of undiagnosed NCDs, while the positive indices for ethnicity (0.050 and 0.049) indicate that ethnic minorities consistently faced higher unmet needs than the Kinh/Hoa majority. A similar pattern was observed in education: the significant concentration of unmet needs among those with lower education in 2015 (ECI: -0.070) was effectively eliminated by 2021 (ECI: -0.003), a change that was appeared reduced.

The distribution of overall unmet needs for NCD treatment across the three diseases showed similar patterns of inequality, favoring the advantaged, with no statistically significant structural changes over the six-year period. Unmet treatment needs remained concentrated among the poor (ECI: -0.053 in 2015; -0.034 in 2021) and ethnic minorities (ECI: 0.025 in 2015; 0.037 in 2021). While the data suggests a numerical reduction in educational inequality, moving from a concentration of unmet needs among the less educated in 2015 (ECI: -0.058) to a near-zero index in 2021 (ECI: 0.003) Table 2, the p-value for the change was 0.8, indicating that, unlike diagnosis, the apparent improvement in treatment equity for education groups was not statistically significant.

**Table 2:** Inequalities in Unmet Needs for NCDs in Viet Nam measured by ECI: Data from STEPS 2015 (n = 1300) and 2021 (n = 2276).

*Variables*	*ECI 2015 (95% CI)*	*ECI 2021 (95% CI)*
Unmet need for diagnosis by SES	-0.11 (-0.16; -0.06)	-0.06 (-0.09; -0.02)
Unmet need for diagnosis by ethnicity	0.05 (0.02; 0.08)	0.05 (0.03; 0.07)
Unmet need for diagnosis by education	-0.07 (-0.12; -0.02)	-0.00 (-0.04; 0.03)
Unmet need for treatment by SES	-0.05 (-0.09; -0.02)	-0.03 (-0.06; -0.01)
Unmet need for treatment by ethnicity	0.03 (0.00; 0.05)	0.04 (0.02; 0.06)
Unmet need for treatment by education	-0.06 (-0.09; -0.02)	0.003 (-0.03; 0.03)

*Note: ECI, Erreygers-normalized Concentration Index; SES, Socioeconomic status.*

Table 3 provides the ECIs estimated separately for each disease. Across all three conditions, socioeconomic and ethnic inequalities were most pronounced for HTN and dyslipidemia, while DM showed minimal gradients. For diagnosis, significant pro-rich inequalities were observed for HTN and dyslipidemia in both 2015 and 2021, particularly for dyslipidemia, where the ECI improved from -0.18 to -0.08 but remained substantial. Ethnic inequalities were also consistent and significant for HTN and dyslipidemia in both years (e.g., ECI for dyslipidemia diagnosis: 0.04 in 2015 to 0.05 in 2021). Educational inequalities were evident in 2015 for dyslipidemia and HTN, but largely disappeared by 2021. For treatment, similar patterns emerged: SES-related inequalities persisted for HTN and dyslipidemia, though they narrowed over time, whereas DM showed no statistically significant inequalities across any domain. Ethnic disparities in treatment were strongest for HTN (ECI: 0.07 in 2015 to 0.10 in 2021) and emerged for dyslipidemia by 2021. Overall, inequalities were largest for dyslipidemia, moderate for HTN, and negligible for DM, with the most persistent disadvantages faced by ethnic minority groups.

**Table 3:** Inequalities in Unmet Needs for hypertension, diabetes, and dyslipidemia in Viet Nam measured by ECI: Data from STEPS 2015 (n = 1300) and 2021 (n = 2276).

*Condition*	*Domain*	*2015* *ECI (95% CI)*	*2021* *ECI (95% CI)*
***Diagnosis***			
Hypertension	SES	−0.15 (−0.23, −0.07)*	−0.07 (−0.13, −0.01)*
	Ethnicity	+0.08 (+0.02, +0.14)*	+0.07 (+0.03, +0.11)*
	Education	−0.07 (−0.15, +0.01)	+0.01 (−0.05, +0.07)
Diabetes	SES	−0.13 (−0.31, +0.05)	−0.05 (−0.17, +0.07)
	Ethnicity	+0.07 (−0.03, +0.17)	+0.06 (−0.02, +0.14)
	Education	−0.02 (−0.20, +0.16)	+0.06 (−0.06, +0.18)
Cholesterol	SES	−0.18 (−0.24, −0.12)*	−0.08 (−0.12, −0.04)*
	Ethnicity	+0.04 (+0.001, +0.08)*	+0.05 (+0.03, +0.07)*
	Education	−0.12 (−0.18, −0.06)*	−0.01 (−0.05, +0.03)
***Treatment***			
Hypertension	SES	−0.15 (−0.21, −0.09)*	−0.08 (−0.14, −0.02)*
	Ethnicity	+0.07 (+0.03, +0.11)*	+0.10 (+0.06, +0.14)*
	Education	−0.08 (−0.14, −0.02)*	−0.01 (−0.07, +0.05)
Diabetes	SES	−0.08 (−0.26, +0.10)	−0.04 (−0.16, +0.08)
	Ethnicity	+0.06 (−0.04, +0.16)	+0.05 (−0.01, +0.11)
	Education	+0.02 (−0.16, +0.20)	+0.08 (−0.04, +0.20)
Cholesterol	SES	−0.06 (−0.10, −0.02)*	−0.03 (−0.05, −0.01)*
	Ethnicity	+0.01 (−0.01, +0.03)	+0.02 (0.00, +0.04)
	Education	−0.07 (−0.11, −0.03)*	+0.01 (−0.01, +0.03)

Note: *p < 0.05; *ECI, Erreygers-normalized Concentration Index; SES, Socioeconomic status.*

### Absolute and Relative Inequality in unmet NCDs diagnosis needs, and unmet NCDs treatment needs: Slope Index (SII) and Relative Index (RII)

In 2015, the unmet need for NCD diagnosis was significantly higher among minority ethnic groups compared with Kinh/Hoa (SII = 0.209, 95% CI: 0.083-0.336, p < 0.05), indicating that the prevalence of unmet need was approximately 21 percentage points higher in minority groups (Table 4). The relative difference (RII = 1.29, 95% CI: 1.14-1.46) indicates that minority groups had 1.29 times the prevalence of unmet need compared to Kinh/Hoa. This ethnic gap remained significant in 2021 (SII = 0.184; RII = 1.25), corresponding to an 18 percentage-point higher prevalence and a 1.25 times higher relative prevalence.

**Table 4:** Ethnic, educational, and socioeconomic inequalities measured by SII and RII in Unmet Needs for NCD diagnosis and treatment among Vietnamese Adults: Data from STEPS 2015 (n = 1300) and 2021 (n = 2276).

*Outcome*	*Equity variable*	*2015-SII (Coef, 95% CI)*	*2015-RII (IRR, 95% CI)*	*2021-SII (Coef, 95% CI)*	*2021-RII (IRR, 95% CI)*
Unmet needs in NCDs diagnosis	Ethnicity	0.209 (0.083; 0.336)	1.29 (1.14; 1.46)	0.184 (0.095; 0.273)	1.25 (1.14; 1.36)
	Education	-0.113 (-0.192; -0.034)	0.87 (0.79; 0.96)	-0.005 (-0.064; 0.055)	0.99 (0.92; 1.07)
	SES	-0.173 (-0.251; -0.096)	0.80 (0.73; 0.89)	-0.089 (-0.146; -0.032)	0.90 (0.84; 0.96)
Unmet needs in NCDs treatment	Ethnicity	0.104 (0.010; 0.198)	1.12 (1.03; 1.22)	0.139 (0.067; 0.210)	1.17 (1.10; 1.24)
	Education	-0.094 (-0.152; -0.035)	0.90 (0.85; 0.96)	0.006 (-0.042; 0.054)	1.01 (0.95; 1.06)
	SES	-0.084 (-0.141; -0.026)	0.91 (0.85; 0.97)	-0.053 (-0.100; -0.007)	0.94 (0.90; 0.99)

*Note: SII, Slope Index of Inequality; RII, Relative Index of Inequality; SES, Socioeconomic status.*

For education, unmet diagnostic needs were higher among the least educated in 2015 (SII = -0.113; RII = 0.87, p < 0.05), meaning 11 percentage points lower prevalence and 0.87 times lower relative prevalence in the population with an education level of college/university compared to the population with an education level of primary school or less. By 2021, educational differences were no longer significant (SII = -0.005; RII = 0.99).

For SES, unmet diagnostic needs were higher in lower-SES groups in 2015 (SII = -0.173; RII = 0.80, p < 0.05), corresponding to 17 percent higher prevalence among the lowest-SES group and 0.8 times lower relative prevalence in the highest-SES group. In 2021, the gap narrowed but remained significant (SII = -0.089; RII = 0.90), indicating a 9-percentage point lower prevalence and a 10% lower relative prevalence in the highest-SES group.

For unmet treatment needs, minority ethnic groups had a higher prevalence in 2015 (SII = 0.104; RII = 1.12, p < 0.05), meaning 10 percentage point higher prevalence and 1.12 times higher relative prevalence, which persisted in 2021 (SII = 0.139; RII = 1.17). Educational and SES differences in treatment needs were significant in 2015 (education SII = -0.094; SES SII = -0.084) but became non-significant in 2021 (education SII = 0.006; SES SII = -0.053).

**Fig 2:**
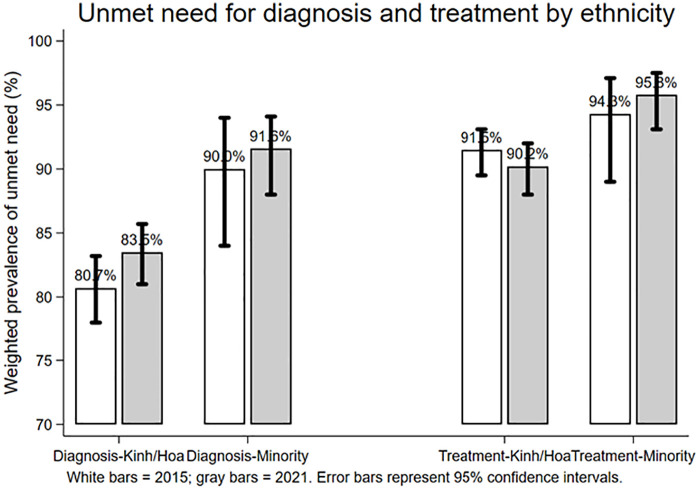
Weighted prevalence of unmet need for NCD diagnosis and treatment by ethnicity, Viet Nam STEPS 2015 and 2021.

Fig 2 illustrates the weighted prevalence of unmet need for diagnosis and treatment by ethnicity in 2015 and 2021. Across both survey years, ethnic minority groups consistently experienced higher unmet needs than the Kinh/Hoa majority. In 2015, unmet diagnosis was 90.0% among ethnic minorities compared with 80.7% among Kinh/Hoa populations; corresponding estimates in 2021 were 91.6% and 83.5%, respectively. Similar patterns were observed for unmet treatment, with prevalence ranging from 94.3% to 95.8% among ethnic minorities compared with 90.2% to 91.5% among Kinh/Hoa populations. These findings visually reinforce the persistent ethnic inequalities identified by the SII and RII analyses.

Sensitivity analyses adjusting for sociodemographic characteristics (age group, sex, residence, education, ethnicity, and SES) generally reduced the magnitude of several inequality estimates, particularly for ethnicity and SES. Nevertheless, the overall interpretation of the findings remained unchanged, with persistent ethnic inequalities and limited educational inequalities observed in 2021. Full results from the adjusted models are provided in S2 Table.

## Discussion

This study provides nationally representative evidence on unmet needs for diagnosis and treatment of HTN, DM, and dyslipidemia in Viet Nam in 2015 and 2021 and documents the social inequalities associated with these gaps. To avoid misinterpretation, we note that the two combined indicators, unmet need for diagnosis and unmet need for treatment, were defined among individuals with any of the three conditions, referring respectively to those who had never been told they had the condition and those who were not taking the corresponding prescribed medication. Overall, unmet need for diagnosis remained extremely high (82% in 2015 and 85% in 2021), and unmet need for treatment was even higher (91% in both years), underscoring substantial and persistent gaps across the NCD care continuum.

Dyslipidemia consistently showed the largest deficits, reflecting its largely asymptomatic nature and the limited availability of routine lipid testing in primary care. The prevalence of unmet treatment needs for DM and HTN was lower in 2021 than in 2015.

Using ECI [13], SII, and RII [14], we identified clear socioeconomic, educational, and ethnic inequalities, though their magnitudes varied by condition. Across diseases, inequalities were most pronounced for HTN and dyslipidemia, while DM showed minimal gradients. Significant pro-rich inequalities in diagnosis were observed for both HTN and dyslipidemia in 2015 and 2021, and ethnic disparities remained consistent across years. Educational inequalities evident in 2015 were no longer apparent in the 2021 survey.. For treatment, SES-related inequalities persisted for HTN and dyslipidemia, although point estimates were generally smaller in the 2021 survey; in contrast, DM showed no significant inequality in any domain. Ethnic disparities in treatment were the strongest for HTN and became evident for dyslipidemia by 2021. Overall, inequalities were largest for dyslipidemia, moderate for HTN, and negligible for DM, with ethnic minority groups experiencing the most persistent disadvantages. Because the sociodemographic composition of the study population differed between survey rounds, particularly with respect to educational attainment, comparisons of inequality estimates over time should be interpreted cautiously. Additional sensitivity analyses adjusting for age, sex, residence, education, ethnicity, and SES generally attenuated the magnitude of several estimates but did not materially alter the overall interpretation of the findings, particularly the persistence of ethnic inequalities and the limited educational inequalities observed in 2021 (S2 Table).

Consistent with RII estimates, ethnic minorities faced 1.29 times higher unmet diagnosis in 2015 and 1.25 times in 2021, and 1.12-1.17 times higher unmet treatment than the Kinh/Hoa majority. Socioeconomic disparities in diagnosis also persisted, while treatment inequalities appeared less pronounced in the 2021 survey. The absence of clear educational inequalities in the 2021 survey may reflect broader improvements in awareness and health literacy. These patterns align with Andersen’s Behavioral Model [10], in which education and ethnicity operate as predisposing factors associated with awareness and help-seeking, while socioeconomic status functions as an enabling factor linked to affordability and access. Persistent ethnic and socioeconomic gradients suggest that structural and cultural barriers exist to both detection and continuity of care [15,16]. Together, these findings illustrate where inequalities in NCD care are most concentrated, specifically at the points of early detection and treatment initiation, an insight highly relevant to LMIC health systems undergoing rapid epidemiological transition [5,6,9,17].

High unmet needs for NCD care have been reported across LMICs, though the magnitude varies by condition. In Nepal, around 50% of adults with HTN remain undiagnosed, indicating substantial gaps in initial detection [6]. In India, 74% of adults with DM are diagnosed, and about 60% of those receive treatment [7], showing attrition along the care continuum. In Thailand, despite universal health coverage and stronger primary care, only 34% of adults with DM are diagnosed, and 33% receive treatment, leaving an estimated 74% with unmet need [9]. These patterns demonstrate that while Viet Nam also faces large diagnosis and treatment gaps, particularly for HTN and DM, the persistence of socioeconomic and ethnic inequities mirrors regional and global evidence of unequal access to chronic disease care [18–20]. Viet Nam’s ethnic disparities are consistent with patterns reported in other LMICs [21]. It is important to note that the 2021 STEPS data collection in Viet Nam was largely completed prior to the major waves of the COVID-19 pandemic. Therefore, our findings essentially reflect a pre-pandemic baseline. Given the profound COVID-19-related disruptions to healthcare systems reported globally and in other LMICs [17,22,23], likely, the substantial unmet needs and ethnic disparities observed in our study have been further exacerbated during and after the pandemic. Future research is needed to assess the true post-pandemic impact on NCD care in Viet Nam.

These findings have several policy implications. First, expanding community-based screening, particularly in ethnic minority regions, is crucial to reducing diagnostic disparities. Second, improving treatment equity requires addressing ongoing medication costs and strengthening continuity of care by making primary care follow-up more accessible. Third, routine equity monitoring using metrics such as ECI, SII, and RII should be integrated into national NCD strategies to identify structural barriers early. Strengthening primary care reforms with culturally and linguistically appropriate approaches will be essential. Importantly, these approaches align with global recommendations for integrating equity-sensitive NCD service coverage into UHC monitoring frameworks, suggesting that Viet Nam’s trajectory offers lessons directly relevant to other LMICs [5].

Key strengths of this study include its use of nationally representative STEPS surveys across two time points, its simultaneous assessment of unmet diagnosis and treatment needs, and its application of multiple complementary inequality measures. Limitations include the cross-sectional design, reliance on self-reported diagnoses and treatments, and the limited number of social determinants examined. In addition, the study examined multiple conditions, outcomes, and inequality measures. Therefore, the findings should be interpreted as providing a comprehensive assessment of inequality patterns rather than as a series of independent hypothesis tests, and some statistically significant findings may be due to chance. Furthermore, the extremely high unmet need for dyslipidemia diagnosis may be partly related to its reliance on biochemical testing, which is less accessible in routine primary care compared to blood pressure screening. This may contribute to higher estimates of unmet need for this specific condition. Despite these limitations, the study provides robust evidence of persistent NCD care inequities in Viet Nam and a replicable framework for monitoring social inequalities in LMICs.

In summary, while income- and education-related inequities appeared less pronounced in the 2021 survey, ethnic disparities remained largely unchanged, underscoring the need for culturally informed and equity-driven strategies to sustain progress toward universal health coverage in Viet Nam. Given similar inequities reported across many LMICs, strengthening equity-oriented primary care systems will be critical for advancing global progress toward SDG 3.4 and achieving truly inclusive UHC.

## Supporting information

S1 Table Missing data among eligible participants included in disease-specific analyses, by survey year. (XLSX)

S2 Table Sensitivity analyses comparing crude and adjusted SII and RII estimates for unmet diagnosis and treatment needs among Vietnamese adults with NCDs, STEPS 2015 and 2021. (XLSX)

S1 File STROBE checklist for cross-sectional studies. (DOCX)
