# Supplementary material for: Persistent socioeconomic and ethnic inequalities in unmet need for NCD diagnosis and treatment in Viet Nam, 2015–2021
Source: PLOS Glob Public Health. 2026 Aug 7;6(8):e0006952. doi: 10.1371/journal.pgph.0006952 (PMC13450777; doi:10.1371/journal.pgph.0006952)
Supplement: Supplementary file 1 [file pgph.0006952.s003.docx]

**STROBE Statement - Checklist of items that should be included in reports of cross-sectional studies**

Completed for manuscript: Persistent socioeconomic and ethnic inequalities in unmet need for NCD diagnosis and treatment in Viet Nam, 2015–2021

Note: Page numbers refer to the corrected manuscript submitted for final technical check. Supporting information files are cited separately as S1 Table and S2 Table. “HTN” = hypertension; “ECI” = Erreygers Concentration Index; “SII” = Slope Index of Inequality; “RII” = Relative Index of Inequality; “SES” = socioeconomic status.

|  | Item No | Recommendation | Reported location / brief details |
| --- | --- | --- | --- |
| **Title and abstract** | 1 | (*a*) Indicate the study’s design with a commonly used term in the title or the abstract | Abstract, p. 1: identifies the study as a repeated cross-sectional analysis of nationally representative WHO STEPS surveys conducted in 2015 and 2021. |
|  |  | (*b*) Provide in the abstract an informative and balanced summary of what was done and what was found | Abstract, pp. 1-2: provides an unstructured, one-paragraph summary of the background, data source, sample sizes, outcomes, inequality measures, principal estimates, and implications. |
| Introduction | | |  |
| Background/rationale | 2 | Explain the scientific background and rationale for the investigation being reported | Introduction, p. 2: describes the global and Viet Nam NCD burden, gaps in diagnosis and treatment, relevance to universal health coverage, and the need to examine inequalities over time. |
| Objectives | 3 | State specific objectives, including any prespecified hypotheses | Introduction, p. 2: states two objectives: to estimate unmet need for diagnosis and treatment of hypertension, diabetes, and dyslipidemia in 2015 and 2021, and to assess socioeconomic, educational, and ethnic inequalities using ECI, SII, and RII. |
| Methods | | |  |
| Study design | 4 | Present key elements of study design early in the paper | Methods: Data Sources and Study Population, p. 3: describes a repeated cross-sectional analysis of nationally representative Viet Nam WHO STEPS surveys from 2015 and 2021. |
| Setting | 5 | Describe the setting, locations, and relevant dates, including periods of recruitment, exposure, follow-up, and data collection | Methods: Data Sources and Study Population, pp. 3-4: describes the Viet Nam setting, national coverage of all 63 provinces and cities, survey years, and completion of 2021 fieldwork before the major COVID-19 waves. Follow-up was not applicable to the cross-sectional design. |
| Participants | 6 | (*a*) Give the eligibility criteria, and the sources and methods of selection of participants | Methods: Data Sources and Study Population, pp. 3-4: gives eligibility criteria and participant selection. Adults aged ≥18 years with Step 3 measurements and at least one eligible condition were included; disease-specific and final analytical sample sizes are reported. |
| Variables | 7 | Clearly define all outcomes, exposures, predictors, potential confounders, and effect modifiers. Give diagnostic criteria, if applicable | Methods: Study Measurements, pp. 4-5: defines unmet diagnosis and unmet treatment for hypertension, diabetes, dyslipidemia, and combined indicators; defines age, sex, residence, ethnicity, education, and SES; and reports diagnostic thresholds. |
| Data sources/ measurement | 8* | For each variable of interest, give sources of data and details of methods of assessment (measurement). Describe comparability of assessment methods if there is more than one group | Methods: Data Sources and Study Population and Study Measurements, pp. 3-5: identifies standardized WHO STEPS questionnaire, physical, and biochemical measurements, including measured blood pressure, fasting glucose, total cholesterol, medication use, and self-reported prior diagnosis. |
| Bias | 9 | Describe any efforts to address potential sources of bias | Methods, pp. 3-6: standardized STEPS procedures, two-stage sampling, fasting requirements, Step 3 weights, PSU/strata adjustment, and assessment of missing data were used to reduce bias. Potential self-report and measurement limitations are discussed in the Discussion, pp. 12-13. |
| Study size | 10 | Explain how the study size was arrived at | Methods: Data Sources and Study Population, pp. 3-4: study size was determined by available Step 3 participants meeting disease-specific eligibility criteria. Step 3 totals, disease-specific samples, and final analytical samples are reported; exclusions were primarily by design. |
| Quantitative variables | 11 | Explain how quantitative variables were handled in the analyses. If applicable, describe which groupings were chosen and why | Methods: Independent Variables and Statistical Analysis, pp. 4-5: reports age categories; education categories; SES derived by PCA and categorized into quintiles; and conversion of ordered social variables to ridit scores for SII and RII estimation. |
| Statistical methods | 12 | (*a*) Describe all statistical methods, including those used to control for confounding | Methods: Statistical Analysis, pp. 4-6: describes survey-weighted prevalence estimates, ECI for binary outcomes, SII from linear probability models, RII from Poisson models with robust standard errors, 95% CIs, significance criteria, and adjusted sensitivity models. |
|  |  | (*b*) Describe any methods used to examine subgroups and interactions | Methods: Statistical Analysis, pp. 5-6; Results, pp. 7-11: analyses were conducted separately by survey year and for overall and disease-specific outcomes across SES, education, and ethnicity. Formal interaction models were not reported. |
|  |  | (*c*) Explain how missing data were addressed | Methods: Statistical Analysis, pp. 5-6; S1 Table (Supporting Information): reports missing data by outcome and year. Six missing education observations in 2015 were retained as a separate category; outcome missingness was 0.08%-6.17%; complete-case analysis was used and multiple imputation was not performed. |
|  |  | (*d*) If applicable, describe analytical methods taking account of sampling strategy | Methods: Data Sources and Study Population and Statistical Analysis, pp. 3-5: describes two-stage systematic random sampling and use of Step 3 weights, PSUs, and strata with svyset psu [pweight=wstep3], strata(stratum). |
|  |  | (*e*) Describe any sensitivity analyses | Methods: Statistical Analysis, pp. 5-6; Results, p. 11; S2 Table (Supporting Information): adjusted SII and RII sensitivity models assessed the influence of sociodemographic composition; conclusions were broadly unchanged. |
| Results | | |  |
| Participants | 13* | (a) Report numbers of individuals at each stage of study-eg numbers potentially eligible, examined for eligibility, confirmed eligible, included in the study, completing follow-up, and analysed | Methods: Data Sources and Study Population, pp. 3-4: reports Step 3 participants (3,074 in 2015; 3,712 in 2021), disease-specific eligible samples, and final analytical samples (1,300 and 2,276). Table 1, pp. 6-7, describes the final samples. |
|  |  | (b) Give reasons for non-participation at each stage | Methods: Data Sources and Study Population, pp. 3-4: explains that the reduction from Step 3 participants to the analytical sample reflected disease-specific eligibility rather than missing data. Survey weights account for non-response in the original surveys. |
|  |  | (c) Consider use of a flow diagram | No flow diagram was included. Participant numbers, eligibility criteria, and reasons for analytical exclusion are reported in Methods, pp. 3-4, and final sample characteristics are reported in Table 1, pp. 6-7. |
| Descriptive data | 14* | (a) Give characteristics of study participants (eg demographic, clinical, social) and information on exposures and potential confounders | Table 1, pp. 6-7: reports age group, education, sex, residence, ethnicity, and SES by survey year for adults with at least one eligible chronic condition. |
|  |  | (b) Indicate number of participants with missing data for each variable of interest | Methods: Statistical Analysis, pp. 5-6, and S1 Table (Supporting Information): report the number and percentage of missing observations for disease-specific diagnosis and treatment outcomes by survey year. |
| Outcome data | 15* | Report numbers of outcome events or summary measures | Results, pp. 7-11: Fig 1 reports weighted prevalence by condition; Fig 2 reports prevalence by ethnicity; Tables 2-4 report ECI, SII, and RII estimates with 95% CIs. |
| Main results | 16 | (*a*) Give unadjusted estimates and, if applicable, confounder-adjusted estimates and their precision (eg, 95% confidence interval). Make clear which confounders were adjusted for and why they were included | Results, pp. 7-11: primary prevalence and inequality estimates are reported with 95% CIs in Fig 1, Fig 2, and Tables 2-4. Methods, pp. 5-6, and S2 Table report adjusted sensitivity models and covariates. |
|  |  | (*b*) Report category boundaries when continuous variables were categorized | Methods: Data Sources and Study Population and Independent Variables, pp. 3-5: reports diagnostic thresholds, age categories, education categories, and SES quintile boundaries. |
|  |  | (*c*) If relevant, consider translating estimates of relative risk into absolute risk for a meaningful time period | Not applicable to this repeated cross-sectional study. Absolute prevalence and SII percentage-point differences are reported alongside relative RII estimates. |
| Other analyses | 17 | Report other analyses done-eg analyses of subgroups and interactions, and sensitivity analyses | Results, pp. 7-11, and S2 Table (Supporting Information): reports disease-specific and ethnicity-specific analyses and adjusted sensitivity analyses. Formal interaction analyses were not conducted. |
| Discussion | | |  |
| Key results | 18 | Summarise key results with reference to study objectives | Discussion, pp. 11-13: summarizes the high unmet need for diagnosis and treatment, the particularly large gaps in dyslipidemia management, and persistent socioeconomic and ethnic inequalities in relation to the study objectives. |
| Limitations | 19 | Discuss limitations of the study, taking into account sources of potential bias or imprecision. Discuss both direction and magnitude of any potential bias | Discussion, pp. 12-13: discusses the cross-sectional design, self-reported diagnoses and treatments, limited social determinants, multiple analyses, and possible inflation of dyslipidemia unmet-need estimates because diagnoses depend on biochemical testing. |
| Interpretation | 20 | Give a cautious overall interpretation of results considering objectives, limitations, multiplicity of analyses, results from similar studies, and other relevant evidence | Discussion, pp. 11-13: provides a cautious interpretation regarding Andersen’s model, regional and global evidence, pre-pandemic timing, sensitivity analyses, and study limitations. |
| Generalisability | 21 | Discuss the generalisability (external validity) of the study results | Discussion, pp. 12-13: notes the national representativeness of STEPS data and the relevance of the findings for Viet Nam and other LMICs pursuing equity-oriented primary care and universal health coverage. |
| Other information | | |  |
| Funding | 22 | Give the source of funding and the role of the funders for the present study and, if applicable, for the original study on which the present article is based | Financial Disclosure in the submission system: the authors received no specific funding for this work; therefore, there was no funder role in study design, data collection, analysis, decision to publish, or manuscript preparation. |

*Give information separately for exposed and unexposed groups.

**Note:** An Explanation and Elaboration article discusses each checklist item and gives methodological background and published examples of transparent reporting. The STROBE checklist is best used in conjunction with this article (freely available on the websites of PLoS Medicine at http://www.plosmedicine.org/, Annals of Internal Medicine at http://www.annals.org/, and Epidemiology at http://www.epidem.com/). Information on the STROBE Initiative is available at www.strobe-statement.org.
