# Supplementary material for: Persistent socioeconomic and ethnic inequalities in unmet need for NCD diagnosis and treatment in Viet Nam, 2015–2021
Source: PLOS Glob Public Health. 2026 Aug 7;6(8):e0006952. doi: 10.1371/journal.pgph.0006952 (PMC13450777; doi:10.1371/journal.pgph.0006952)
Supplement: Supplementary file 3 [file pgph.0006952.s006.docx]

**Overall response:**

We sincerely thank the reviewer for the very insightful and constructive comments. We appreciate the reviewer’s careful assessment of the statistical methods, reporting clarity, and interpretation of the findings. We have revised the manuscript accordingly, with particular attention to survey weighting, missing data, model specification, sample comparability, visualization, interpretation of multiple analyses, and the abstract. Our point-by-point responses are provided below.

### Comment 1

Major
1) please use an appropriate research checklist, e.g. STROBE.

**Response:** Thank you for this request. We have completed the STROBE checklist for cross-sectional studies following the journal’s required template. The checklist has been carefully reviewed against the revised manuscript and uploaded as part of the resubmission package.

We also checked the manuscript to ensure that the relevant STROBE items are adequately reported, including the study design, data sources, study population, variable definitions, statistical methods, handling of missing data, sensitivity analyses, key results, limitations, generalisability, and funding statement.

Comment 2:
2) clarify the use of weights. how derived, where available and what commands were used in stata that account for weighting.

**Response:** Thank you for this helpful comment. We have revised the Methods section to provide additional details regarding survey weighting. The WHO STEPS datasets include survey design variables and sampling weights for each survey component (Steps 1–3). Because our analyses were restricted to participants who completed biochemical assessments, we applied the Step 3 sampling weight (wstep3) provided in the STEPS datasets. These weights were developed by the STEPS survey team to account for differential probabilities of selection, non-response, and post-stratification adjustments, ensuring national representativeness. All analyses accounted for the complex survey design using primary sampling units (PSUs), strata, and Step 3 sampling weights. In Stata, survey settings were specified using:

svyset psu [pweight=wstep3], strata(stratum)

Weighted prevalence estimates and survey-adjusted regression models used to estimate SII and RII were subsequently conducted using the svy: prefix. The relevant information has now been added to the Statistical Analysis section.

Comment 3:
3) clarify how you dealt with missing data. it is extremely unlikely data were complete, and levels of missingness need to be reported for each variable of interest and each model (how many cases dropped). if over 10% of cases are dropped why weren't multiple imputation approaches considered?

**Response:** Thank you for this important comment. We have now added a description of missing data handling to the Methods section and reported the levels of missingness for the variables included in the analyses. We would like to clarify that the reduction from all Step 3 participants to the final analytical sample was primarily due to disease-specific eligibility criteria rather than missing data. Participants were intentionally restricted to those with hypertension, diabetes, and/or dyslipidemia, as these groups constituted the target population for the unmet-need analyses.

Among eligible participants, missingness was low. For sociodemographic variables, only six participants in 2015 had missing information on education; these observations were retained as a separate category (“missing/no information”) and were not excluded from the analyses. For outcome variables, missingness ranged from 0.08% to 6.17%. Specifically, missing diagnosis information ranged from 0.08% to 2.27% across conditions and survey years, while missing treatment information ranged from 0.30% to 6.17%, with the highest level observed for dyslipidemia treatment in 2015 (53/859 participants).

We have added Supplementary Table S1 showing the number and percentage of missing observations for each outcome. Because missingness was low and no variable exceeded 10%, complete-case analysis was considered appropriate and multiple imputation was not performed.

Comment 4:
4) The modelling approach is broadly appropriate but the description lacks sufficient clarity. It is unclear which covariates were included in the main models, how they were selected, and whether all sociodemographic factors were adjusted for simultaneously.

**Response:** Thank you for this helpful comment. We have revised the Statistical Analysis section to clarify the modelling strategy. For each inequality determinant (SES, education, and ethnicity), we first estimated crude SII and RII models. We then fitted adjusted models to account for differences in population composition between survey years. Adjusted models included age group, sex, place of residence, education, ethnicity, and SES, where appropriate. Covariates were selected a priori based on their established associations with healthcare access and NCD management and were included to reduce potential confounding. The revised Methods section now explicitly describes the covariates included in both crude and adjusted models.

Comment 5:
5) The analysis compares inequality measures across 2015 and 2021, but does not formally test whether these differences are statistically significant. A pooled model including interaction terms between time and the inequality variables (e.g. SES, ethnicity) would allow a more robust assessment of whether inequalities have widened or narrowed over time.

**Response**

Thank you for this insightful suggestion. We agree that pooled models that include interaction terms between survey year and inequality indicators would provide a formal statistical test of changes in inequality over time. However, the primary objective of this study was to estimate and compare survey-year-specific prevalence and inequality measures (ECI, SII, and RII) using nationally representative STEPS surveys from 2015 and 2021. Accordingly, comparisons between years were intended to describe changes in the magnitude and direction of inequalities rather than formally test interactions.

We also note that the study simultaneously examined multiple NCD conditions, diagnosis and treatment outcomes, and several inequality measures. Incorporating pooled interaction models for each outcome and inequality dimension would substantially expand the analytical framework and address a related, but distinct, research question. We have therefore retained the year-specific analyses, clarified their interpretation in the manuscript, and acknowledged the absence of formal interaction testing as a limitation and an area for future research.

Comment 6:

6) The comparability of the 2015 and 2021 samples is unclear, particularly given the substantial differences in educational composition shown in Table 1. While the manuscript notes the use of survey weights, it is not clear whether these adequately account for these shifts, especially within the regression analyses. This raises concerns about whether changes (or lack thereof) in inequalities over time may partly reflect differences in sample composition rather than true population trends. More information is needed on the weighted samples and demonstration that after weighting the samples are comparable in terms of key variables.

**Response:** Thank you for this important comment. We agree that differences in sociodemographic composition between the 2015 and 2021 samples, particularly educational attainment, should be more clearly addressed. We have revised the Methods section to clarify that all analyses accounted for the complex survey design using Step 3 sampling weights, primary sampling units, and strata provided in the WHO STEPS datasets. These weights were used to generate nationally representative estimates for each survey year.

We would also like to clarify the role of the crude SII and RII estimates in our analysis. The primary analyses were based on crude SII and RII estimates because these indices are conventionally used in health inequality monitoring to quantify population-level absolute and relative inequalities. Therefore, they were retained as the main inequality measures in the manuscript. However, in response to the reviewer’s concern, we conducted additional survey-weighted sensitivity analyses adjusting for key sociodemographic factors, including age group, sex, residence, education, ethnicity, and socioeconomic status, as appropriate.

The adjusted analyses generally attenuated the magnitude of several estimates, particularly for ethnicity and SES, indicating that part of the observed inequality may be related to differences in sociodemographic composition. However, the overall interpretation of the findings remained broadly similar: ethnic inequalities, particularly in 2021, persisted after adjustment, while educational inequalities were limited in 2021. These adjusted results are now presented in Supplementary Table S2 for transparency, but they are treated as sensitivity analyses rather than replacing the primary population-level inequality estimates.

We have also added a short discussion acknowledging that differences in population composition across survey years may influence the interpretation of changes in inequality over time and that the adjusted analyses should be considered when interpreting temporal patterns.

Comment 7:
7) the paper lacks severely in visualisation of the results.

**Response:** Thank you for this valuable suggestion. We agree that visual presentation can improve the accessibility and interpretation of the findings. The manuscript already included a figure presenting the prevalence of unmet diagnosis and treatment across NCD conditions and survey years. In response to the reviewer's comment, we have added a new figure (Figure 2) illustrating the weighted prevalence of unmet need for diagnosis and treatment by ethnicity in 2015 and 2021, including 95% confidence intervals. We selected ethnicity because it represented the most consistent and persistent source of inequality across outcomes and survey years, as demonstrated by the ECI, SII, and RII analyses. This figure complements the tabulated results and facilitates interpretation of the magnitude of ethnic disparities in NCD care.

Comment 8:
8) there are perhaps too many outcomes and analyses, hence findings need to be treated as exploratory, due to type-I error inflation.

**Response:** Thank you for this important observation. We agree that the study examined multiple conditions, outcomes, and inequality measures, which increases the possibility that some statistically significant findings may occur by chance. However, our primary objective was not to test a large number of independent hypotheses, but rather to provide a comprehensive assessment of unmet needs and social inequalities in NCD diagnosis and treatment using complementary inequality measures (ECI, SII, and RII) that capture different dimensions of inequality.

To address this concern, we have revised the Discussion section to emphasize a more cautious interpretation of the findings and added a limitation acknowledging the potential for type-I error inflation arising from multiple analyses. We also note that the major conclusions were supported by consistent patterns across multiple inequality measures and across both survey rounds, which increases confidence in the robustness of the overall findings.

Comment 9:
9) the abstract needs to better reflect the study e.g. the GLMs are not mentioned and there are no results from the models that i could see. The size of the effects need to be considered, not just statistical significance.

**Response:** Thank you for this helpful suggestion. We have revised the abstract to better reflect both the analytical approach and the key findings of the study. Specifically, we clarified that absolute and relative inequalities were assessed using SII and RII estimated from linear and Poisson regression models, respectively. We also added quantitative results from the inequality analyses, including RII estimates and 95% confidence intervals, to provide information on the magnitude of ethnic and socioeconomic inequalities rather than relying solely on statements of statistical significance. These revisions provide a more balanced summary of both the prevalence of unmet needs and the observed patterns of inequality.

Comment 10:

10) Clarify "ridit" is an acronym

**Response:** Thank you for this helpful comment. We have clarified the meaning of ridit in the Statistical Analysis section. Specifically, ridit scores (Relative to an Identified Distribution) were used to represent each participant's relative position in the cumulative distribution of the ordered social variable and were subsequently used to estimate SII and RII
