# Supplementary material for: Persistent socioeconomic and ethnic inequalities in unmet need for NCD diagnosis and treatment in Viet Nam, 2015–2021
Source: PLOS Glob Public Health. 2026 Aug 7;6(8):e0006952. doi: 10.1371/journal.pgph.0006952 (PMC13450777; doi:10.1371/journal.pgph.0006952)
Supplement: Supplementary file 4 [file pgph.0006952.s005.docx]

***Dear Editor and Reviewers,***

We would like to thank the Editor and the Reviewers for taking the time to review our manuscript entitled *"Persistent socioeconomic and ethnic inequalities in unmet need for NCD diagnosis and treatment in Vietnam, 2015-2021"*. We sincerely appreciate the positive assessment of our study and the highly constructive comments provided.

We have carefully considered all the feedback and have thoroughly revised the manuscript accordingly. We believe that these revisions have significantly improved the clarity, rigor, and quality of our paper.

Below, please find our point-by-point responses to each of the reviewers' comments. The corresponding changes in the revised manuscript have been highlighted using the track changes feature.

Sincerely,

Quyen Bui

--------------------------

Reviewers' comments:

Reviewer #1:

Comment 1: General Comment
This manuscript addresses an important and timely public health issue by examining socioeconomic and ethnic inequalities in unmet need for diagnosis and treatment of major noncommunicable diseases in Vietnam using nationally representative data. The study is methodologically sound, well written, and policy relevant. The use of repeated STEPS surveys and multiple inequality measures (ECI, SII, RII) strengthens the contribution. However, several issues need to be addressed before further evaluation.

Response: We sincerely thank the reviewer for the positive assessment of our study's significance and methodological rigor. We have carefully addressed all the specific points raised to improve the clarity and quality of the manuscript.

Comment 2: Clarification of age eligibility. The manuscript states that STEPS includes adults aged ≥15 years, while biochemical measurements were collected among those aged ≥18 years. The authors should explicitly clarify in the Methods section that the analytical sample was restricted to participants with available biochemical data (≥18 years) to avoid confusion.

Response: We agree with the reviewer. We have revised the "Data Sources" section to explicitly state that, while the general STEPS survey includes individuals aged 15, our analysis is restricted to adults aged 18 years and older because biochemical data (required to define unmet needs) were available only for this subgroup. The manuscript has been updated.

Comment 3: Terminology in inequality interpretation. In the Statistical Analysis section, the description of the Slope Index of Inequality refers to “higher participation” instead of “higher unmet need.” This wording should be corrected to accurately reflect the outcome under study.

Response: Thank you for pointing out this inconsistency. We have corrected the terminology throughout the Statistical Analysis section. We now use "higher unmet need" to ensure the interpretation aligns with our study outcomes.

Comment 4: Table numbering. Two tables are labelled as Table 3. Table numbering should be corrected to ensure consistency and avoid reader confusion.

Response: We apologize for this oversight. The tables have been renumbered sequentially. The original second "Table 3" (showing SII and RII results) is now Table 4.

Comment 5: Data Sources and Study Population. The authors state that “ Diabetes (DTD): fasting blood glucose ≥7.0 mmol/L, or current use of insulin or other glucose-lowering medication (n = 132 in 2015; n = 305 in 2021” ….what about does that did not fast?

Response: We have clarified this in the Methods. Only participants who self-reported fasting for at least 8 hours prior to blood sampling were included in the definition of diabetes prevalence and in the estimation of unmet need for diabetes. Participants who did not meet the fasting requirement were excluded from diabetes-specific analyses

Comment 6: Secondly the authors should clearly state the selection criteria, since the current version is a bit confusing

Response: We thank the reviewer for highlighting the need for greater clarity regarding the selection criteria. We have substantially revised the *Data Sources and Study Population* section to more clearly describe the analytic sample. Specifically, we now state that the analysis was restricted to adults aged ≥18 years who completed Step 3 measurements and were identified as having at least one of the three target conditions (hypertension, diabetes, or dyslipidemia), defined based on objective measurements and/or current use of condition-specific medication. We also clarified that, for diabetes classification, only participants who reported fasting for ≥8 hours prior to blood sampling were eligible for inclusion based on glucose measurements. Finally, we explicitly report the number of participants meeting these criteria in each survey year and note that the final analytic sample accounts for overlapping conditions (multimorbidity). These revisions improve transparency and eliminate ambiguity regarding the study population

Comment 7: Independent Variables and Table 1: The age category <25 does not align with what is written under methodology, and suggests that the study has included participants aged below 15 years, considered 15/18-24. Please correct accordingly

Response: Yes, that is correct. Since the sample is restricted to those ≥18 years, the "<25" group refers to the 18–24 age range. We have updated the label in Table 1 for clarity.

Comment 8: Consistency in outcome terminology. Terms such as “diagnosed by SES” and “unmet need for diagnosis” are used interchangeably. The authors should standardize terminology throughout the text, tables, and figure captions to consistently use “unmet need for diagnosis” and “unmet need for treatment.”

Response: We have reviewed the entire manuscript and tables to ensure consistent terminology. Labels such as "Diagnosed by SES" in the previous tables have been changed to "Unmet need for diagnosis by SES" to avoid any ambiguity.

Comment 9: Abstract clarity. The abstract would benefit from explicitly stating that unmet needs for diagnosis and treatment were analyzed as separate outcomes. In addition, numerical detail in the Results section of the abstract could be slightly reduced to improve readability.

Response: We have restructured the Abstract. We now explicitly state that diagnosis and treatment needs were treated as two distinct outcomes. We have also removed redundant numerical data to make the key findings more prominent.

Comment 10: Ethical considerations: Please add the reference number

Response: We have added the ethical approval reference number from the Institutional Review Board (IRB) at Hanoi University of Public Health.

Comment 11: Figure 1 caption. The caption for Figure 1 does not clearly specify the study population or data source. Please indicate that the figure is based on adults with measured conditions from the 2015 and 2021 Vietnam STEPS surveys. Correct labelling of figures, please check Plos guidelines

Response: The caption for Figure 1 has been updated to include the source (STEPS 2015 & 2021) and the specific population (adults aged ≥18 with measured NCD conditions). We have also ensured the figure meets PLOS formatting requirements.

Comment 12: Causal language: Some statements in the Discussion imply causality (e.g., education “reduced” inequalities). Given the cross-sectional design, causal language should be softened to “was associated with” or “coincided with.”

Response: We agree. Given the cross-sectional nature of the STEPS surveys, we have replaced causal verbs like "reduced" or "impacted" with "was associated with," "correlated with," or "contributed to."

Comment 13: COVID-19 contextualization. The manuscript suggests that COVID-19 may have influenced unmet needs in 2021. The authors should acknowledge that pandemic-related disruptions were not directly measured and interpret these findings cautiously.

Response: We thank the reviewer for this insightful comment. We completely agree that pandemic-related disruptions were not directly measured. In fact, field data collection for the 2021 STEPS survey was largely completed before the major COVID-19 outbreaks and subsequent lockdowns in Vietnam. Therefore, our 2021 data actually reflects a pre-pandemic baseline. To avoid any misinterpretation, we have revised the Discussion. We removed the implication that COVID-19 directly influenced our 2021 estimates. Instead, we reframed this section to emphasize that our findings serve as a pre-pandemic baseline, and we cautiously speculate, supported by literature, that these already high unmet needs may have worsened subsequently due to the pandemic.

Comment 14: Measurement-related limitation. Dyslipidemia diagnosis relies heavily on biochemical testing, which may inflate unmet need estimates relative to other NCDs. This should be acknowledged explicitly as a study limitation.

Response: We thank the reviewer for this important observation. We have explicitly incorporated this issue into the Limitations section. Specifically, we note that dyslipidemia diagnosis relies primarily on biochemical testing, which is less routinely available in primary care compared with blood pressure measurement for hypertension. Because dyslipidemia is often asymptomatic and screening requires laboratory testing, this diagnostic modality difference may lead to comparatively higher estimates of unmet need for diagnosis relative to other NCDs.
