# Supplementary material for: Persistent socioeconomic and ethnic inequalities in unmet need for NCD diagnosis and treatment in Viet Nam, 2015–2021
Source: PLOS Glob Public Health. 2026 Aug 7;6(8):e0006952. doi: 10.1371/journal.pgph.0006952 (PMC13450777; doi:10.1371/journal.pgph.0006952)
Supplement: Peer review history

# Peer review: Decision Letter 0

**DOI:** 10.1371/journal.pgph.0006952.r001

**Authors:** Helen Howard

15 Feb 2026

PGPH-D-25-03848

Persistent socioeconomic and ethnic inequalities in unmet need for NCD diagnosis and treatment in Vietnam, 2015–2021

PLOS Global Public Health

Dear Dr. Bui,

Thank you for submitting your manuscript to PLOS Global Public Health. After careful consideration, we feel that it has merit but does not fully meet PLOS Global Public Health’s publication criteria as it currently stands. Therefore, we invite you to submit a revised version of the manuscript that addresses the points raised during the review process.

Please note that we have only been able to secure a single reviewer to assess your manuscript. We are issuing a decision on your manuscript at this point to prevent further delays in the evaluation of your manuscript. Please be aware that the editor who handles your revised manuscript might find it necessary to invite additional reviewers to assess this work once the revised manuscript is submitted. However, we will aim to proceed on the basis of this single review if possible.

Could you please carefully revise the manuscript to address all comments raised?

Please submit your revised manuscript by Mar 30 2026 11:59PM. If you will need more time than this to complete your revisions, please reply to this message or contact the journal office at globalpubhealth@plos.org. When you're ready to submit your revision, log on to https://www.editorialmanager.com/pgph/ and select the 'Submissions Needing Revision' folder to locate your manuscript file.

Please include the following items when submitting your revised manuscript:

- A letter that responds to each point raised by the editor and reviewer(s). You should upload this letter as a separate file labeled 'Response to Reviewers'.
- A marked-up copy of your manuscript that highlights changes made to the original version. You should upload this as a separate file labeled 'Revised Manuscript with Track Changes'.
- An unmarked version of your revised paper without tracked changes. You should upload this as a separate file labeled 'Manuscript'.

Guidelines for resubmitting your figure files are available below the reviewer comments at the end of this letter.

We look forward to receiving your revised manuscript.

Kind regards,

Helen Howard

Staff Editor

PLOS Global Public Health

Journal Requirements:

If the reviewer comments include a recommendation to cite specific previously published works, please review and evaluate these publications to determine whether they are relevant and should be cited. There is no requirement to cite these works unless the editor has indicated otherwise.

Additional Editor Comments (if provided):

[Note: HTML markup is below. Please do not edit.]

Reviewers' comments:

Reviewer's Responses to Questions

**Comments to the Author**

1. Does this manuscript meet PLOS Global Public Health’s publication criteria? Is the manuscript technically sound, and do the data support the conclusions? The manuscript must describe methodologically and ethically rigorous research with conclusions that are appropriately drawn based on the data presented.

Reviewer #1: Yes

**********

2. Has the statistical analysis been performed appropriately and rigorously?

Reviewer #1: Yes

**********

3. Have the authors made all data underlying the findings in their manuscript fully available (please refer to the Data Availability Statement at the start of the manuscript PDF file)?

The PLOS Data policy requires authors to make all data underlying the findings described in their manuscript fully available without restriction, with rare exception. The data should be provided as part of the manuscript or its supporting information, or deposited to a public repository. For example, in addition to summary statistics, the data points behind means, medians and variance measures should be available. If there are restrictions on publicly sharing data—e.g. participant privacy or use of data from a third party—those must be specified.

Reviewer #1: No

**********

4. Is the manuscript presented in an intelligible fashion and written in standard English?

PLOS Global Public Health does not copyedit accepted manuscripts, so the language in submitted articles must be clear, correct, and unambiguous. Any typographical or grammatical errors should be corrected at revision, so please note any specific errors here.

Reviewer #1: Yes

**********

5. Review Comments to the Author

Please use the space provided to explain your answers to the questions above. You may also include additional comments for the author, including concerns about dual publication, research ethics, or publication ethics. (Please upload your review as an attachment if it exceeds 20,000 characters)

Reviewer #1: General Comment

This manuscript addresses an important and timely public health issue by examining socioeconomic and ethnic inequalities in unmet need for diagnosis and treatment of major noncommunicable diseases in Vietnam using nationally representative data. The study is methodologically sound, well written, and policy relevant. The use of repeated STEPS surveys and multiple inequality measures (ECI, SII, RII) strengthens the contribution. However, several issues need to be addressed before further evaluation.

Major Comments

Clarification of age eligibility

The manuscript states that STEPS includes adults aged ≥15 years, while biochemical measurements were collected among those aged ≥18 years.

The authors should explicitly clarify in the Methods section that the analytical sample was restricted to participants with available biochemical data (≥18 years) to avoid confusion.

1. Terminology in inequality interpretation

In the Statistical Analysis section, the description of the Slope Index of Inequality refers to “higher participation” instead of “higher unmet need.” This wording should be corrected to accurately reflect the outcome under study.

2. Table numbering

Two tables are labelled as Table 3. Table numbering should be corrected to ensure consistency and avoid reader confusion.

3. Data Sources and Study Population

The authors state that “ Diabetes (DTD): fasting blood glucose ≥7.0 mmol/L, or current use of insulin or other glucose-lowering medication (n = 132 in 2015; n = 305 in 2021” ….what about does that did not fast?

Secondly the authors should clearly state the selection criteria, since the current version is a bit confusing

4. Independent Variables and Table 1

The age category <25 does not align with what is written under methodology, and suggests that the study has included participants aged below 15 years, considered 15/18-24. Please correct accordingly

Minor Comments

Consistency in outcome terminology

Terms such as “diagnosed by SES” and “unmet need for diagnosis” are used interchangeably.

The authors should standardize terminology throughout the text, tables, and figure captions to consistently use “unmet need for diagnosis” and “unmet need for treatment.”

Abstract clarity

The abstract would benefit from explicitly stating that unmet needs for diagnosis and treatment were analyzed as separate outcomes. In addition, numerical detail in the Results section of the abstract could be slightly reduced to improve readability.

Ethical considerations

Please add the reference number

Figure 1 caption

The caption for Figure 1 does not clearly specify the study population or data source.

Please indicate that the figure is based on adults with measured conditions from the 2015 and 2021 Vietnam STEPS surveys.

Correct labelling of figures, please check Plos guidelines

Causal language

Some statements in the Discussion imply causality (e.g., education “reduced” inequalities).

Given the cross-sectional design, causal language should be softened to “was associated with” or “coincided with.”

COVID-19 contextualization

The manuscript suggests that COVID-19 may have influenced unmet needs in 2021.

The authors should acknowledge that pandemic-related disruptions were not directly measured and interpret these findings cautiously.

Measurement-related limitation

Dyslipidemia diagnosis relies heavily on biochemical testing, which may inflate unmet need estimates relative to other NCDs. This should be acknowledged explicitly as a study limitation.

**********

6. PLOS authors have the option to publish the peer review history of their article (what does this mean?). If published, this will include your full peer review and any attached files.

**Do you want your identity to be public for this peer review?**  If you choose “no”, your identity will remain anonymous but your review may still be made public.

For information about this choice, including consent withdrawal, please see our Privacy Policy.

Reviewer #1: **Yes:** Cairo Bruce Ntimana

**********

[NOTE: If reviewer comments were submitted as an attachment file, they will be attached to this email and accessible via the submission site. Please log into your account, locate the manuscript record, and check for the action link "View Attachments". If this link does not appear, there are no attachment files.]

-->Figure Resubmissions:

While revising your submission, we strongly recommend that you use PLOS’s NAAS tool (https://ngplosjournals.pagemajik.ai/artanalysis) to test your figure files. NAAS can convert your figure files to the TIFF file type and meet basic requirements (such as print size, resolution), or provide you with a report on issues that do not meet our requirements and that NAAS cannot fix.

After uploading your figures to PLOS’s NAAS tool - https://ngplosjournals.pagemajik.ai/artanalysis, NAAS will process the files provided and display the results in the "Uploaded Files" section of the page as the processing is complete. If the uploaded figures meet our requirements (or NAAS is able to fix the files to meet our requirements), the figure will be marked as "fixed" above. If NAAS is unable to fix the files, a red "failed" label will appear above. When NAAS has confirmed that the figure files meet our requirements, please download the file via the download option, and include these NAAS processed figure files when submitting your revised manuscript.

# Author comment: Author response to Decision Letter 1

**DOI:** 10.1371/journal.pgph.0006952.r002

25 Feb 2026

Attachment Submitted filename: Reviewers 1.docx

# Peer review: Decision Letter 1

**DOI:** 10.1371/journal.pgph.0006952.r003

**Authors:** Alejandro Torrado Pacheco

12 Jun 2026

PGPH-D-25-03848R1

Persistent socioeconomic and ethnic inequalities in unmet need for NCD diagnosis and treatment in Vietnam, 2015–2021

PLOS Global Public Health

Dear Dr. Bui,

Thank you for submitting your manuscript to PLOS Global Public Health. After careful consideration, we feel that it has merit but does not fully meet PLOS Global Public Health’s publication criteria as it currently stands. Therefore, we invite you to submit a revised version of the manuscript that addresses the points raised during the review process.

The manuscript has been evaluated by three reviewers and their comments are available below. Please address all comments carefully.

Please submit your revised manuscript by Aug 10 2026 11:59PM. If you will need more time than this to complete your revisions, please reply to this message or contact the journal office at globalpubhealth@plos.org. When you're ready to submit your revision, log on to https://www.editorialmanager.com/pgph/ and select the 'Submissions Needing Revision' folder to locate your manuscript file.

Please include the following items when submitting your revised manuscript:

- A letter that responds to each point raised by the editor and reviewer(s). You should upload this letter as a separate file labeled 'Response to Reviewers'.
- A marked-up copy of your manuscript that highlights changes made to the original version. You should upload this as a separate file labeled 'Revised Manuscript with Track Changes'.
- An unmarked version of your revised paper without tracked changes. You should upload this as a separate file labeled 'Manuscript'.

Guidelines for resubmitting your figure files are available below the reviewer comments at the end of this letter.

As the corresponding author, your ORCID iD is verified in the submission system and will appear in the published article. PLOS supports the use of ORCID, and we encourage all coauthors to register for an ORCID iD and use it as well. Please encourage your coauthors to verify their ORCID iD within the submission system before final acceptance, as unverified ORCID iDs will not appear in the published article. *Only*  the individual author can complete the verification step; PLOS staff *cannot*  verify ORCID iDs on behalf of authors.

We look forward to receiving your revised manuscript.

Kind regards,

Alejandro Torrado Pacheco, PhD

PLOS Editor

PLOS Global Public Health

**Journal Requirements:**

If the reviewer comments include a recommendation to cite specific previously published works, please review and evaluate these publications to determine whether they are relevant and should be cited. There is no requirement to cite these works unless the editor has indicated otherwise.

**Additional Editor Comments (if provided):**

[Note: HTML markup is below. Please do not edit.]

Reviewers' comments:

Reviewer's Responses to Questions

**Comments to the Author**

1. If the authors have adequately addressed your comments raised in a previous round of review and you feel that this manuscript is now acceptable for publication, you may indicate that here to bypass the “Comments to the Author” section, enter your conflict of interest statement in the “Confidential to Editor” section, and submit your "Accept" recommendation.

Reviewer #1: All comments have been addressed

Reviewer #2: All comments have been addressed

Reviewer #3: (No Response)

**********

2. Does this manuscript meet PLOS Global Public Health’s publication criteria? Is the manuscript technically sound, and do the data support the conclusions? The manuscript must describe methodologically and ethically rigorous research with conclusions that are appropriately drawn based on the data presented.

Reviewer #1: Yes

Reviewer #2: Yes

Reviewer #3: Yes

**********

3. Has the statistical analysis been performed appropriately and rigorously?

Reviewer #1: Yes

Reviewer #2: Yes

Reviewer #3: Yes

**********

4. Have the authors made all data underlying the findings in their manuscript fully available (please refer to the Data Availability Statement at the start of the manuscript PDF file)?

The PLOS Data policy requires authors to make all data underlying the findings described in their manuscript fully available without restriction, with rare exception. The data should be provided as part of the manuscript or its supporting information, or deposited to a public repository. For example, in addition to summary statistics, the data points behind means, medians and variance measures should be available. If there are restrictions on publicly sharing data—e.g. participant privacy or use of data from a third party—those must be specified.

Reviewer #1: Yes

Reviewer #2: Yes

Reviewer #3: Yes

**********

5. Is the manuscript presented in an intelligible fashion and written in standard English?

PLOS Global Public Health does not copyedit accepted manuscripts, so the language in submitted articles must be clear, correct, and unambiguous. Any typographical or grammatical errors should be corrected at revision, so please note any specific errors here.

Reviewer #1: Yes

Reviewer #2: Yes

Reviewer #3: Yes

**********

6. Review Comments to the Author

Please use the space provided to explain your answers to the questions above. You may also include additional comments for the author, including concerns about dual publication, research ethics, or publication ethics. (Please upload your review as an attachment if it exceeds 20,000 characters)

**Reviewer #1:**  The authors have addressed all the comments

**Reviewer #2:**  Since this was a revised version and all the comments have been addressed. So, I don't have any future comments. thanks

**Reviewer #3:**  As the statistical reviewer I will focus on methods and reporting.

Major

1) please use an appropriate research checklist, e.g. STROBE.

2) clarify the use of weights. how derived, where available and what commands were used in stata that account for weighting.

3) clarify how you dealt with missing data. it is extremely unlikely data were complete, and levels of missingness need to be reported for each variable of interest and each model (how many cases dropped). if over 10% of cases are dropped why weren't multiple imputation approaches considered?

4) The modelling approach is broadly appropriate but the description lacks sufficient clarity. It is unclear which covariates were included in the main models, how they were selected, and whether all sociodemographic factors were adjusted for simultaneously.

5) The analysis compares inequality measures across 2015 and 2021, but does not formally test whether these differences are statistically significant. A pooled model including interaction terms between time and the inequality variables (e.g. SES, ethnicity) would allow a more robust assessment of whether inequalities have widened or narrowed over time.

6) The comparability of the 2015 and 2021 samples is unclear, particularly given the substantial differences in educational composition shown in Table 1. While the manuscript notes the use of survey weights, it is not clear whether these adequately account for these shifts, especially within the regression analyses. This raises concerns about whether changes (or lack thereof) in inequalities over time may partly reflect differences in sample composition rather than true population trends. More information is needed on the weighted samples and demonstration that after weighting the samples are comparable in terms of key variables.

7) the paper lacks severely in visualisation of the results.

8) there are perhaps too many outcomes and analyses, hence findings need to be treated as exploratory, due to type-I error inflation.

9) the abstract needs to better reflect the study e.g. the GLMs are not mentioned and there are no results from the models that i could see. The size of the effects need to be considered, not just statistical significance.

Minor

1) clarify "ridit" is an acronym

**********

7. PLOS authors have the option to publish the peer review history of their article (what does this mean?). If published, this will include your full peer review and any attached files.

**Do you want your identity to be public for this peer review?**  If you choose “no”, your identity will remain anonymous but your review may still be made public.

For information about this choice, including consent withdrawal, please see our Privacy Policy.

Reviewer #1: **Yes:** Cairo Bruce Ntimana

Reviewer #2: **Yes:** Virak Sorn

Reviewer #3: No

**********

[NOTE: If reviewer comments were submitted as an attachment file, they will be attached to this email and accessible via the submission site. Please log into your account, locate the manuscript record, and check for the action link "View Attachments". If this link does not appear, there are no attachment files.]

**Figure Resubmissions:**

While revising your submission, we strongly recommend that you use PLOS’s NAAS tool (https://ngplosjournals.pagemajik.ai/artanalysis) to test your figure files. NAAS can convert your figure files to the TIFF file type and meet basic requirements (such as print size, resolution), or provide you with a report on issues that do not meet our requirements and that NAAS cannot fix.

After uploading your figures to PLOS’s NAAS tool - https://ngplosjournals.pagemajik.ai/artanalysis, NAAS will process the files provided and display the results in the "Uploaded Files" section of the page as the processing is complete. If the uploaded figures meet our requirements (or NAAS is able to fix the files to meet our requirements), the figure will be marked as "fixed" above. If NAAS is unable to fix the files, a red "failed" label will appear above. When NAAS has confirmed that the figure files meet our requirements, please download the file via the download option, and include these NAAS processed figure files when submitting your revised manuscript.

# Author comment: Author response to Decision Letter 2

**DOI:** 10.1371/journal.pgph.0006952.r004

15 Jun 2026

Attachment Submitted filename: response to reviewer Jun2026.docx

# Peer review: Decision Letter 2

**DOI:** 10.1371/journal.pgph.0006952.r005

**Authors:** Priyanka Roy

13 Jul 2026

Persistent socioeconomic and ethnic inequalities in unmet need for NCD diagnosis and treatment in Vietnam, 2015–2021

PGPH-D-25-03848R2

Dear Dr. Bui,

We are pleased to inform you that your manuscript 'Persistent socioeconomic and ethnic inequalities in unmet need for NCD diagnosis and treatment in Vietnam, 2015–2021' has been provisionally accepted for publication in PLOS Global Public Health.

Before your manuscript can be formally accepted you will need to complete some formatting changes, which you will receive in a follow up email. A member of our team will be in touch with a set of requests.

Please note that your manuscript will not be scheduled for publication until you have made the required changes, so a swift response is appreciated.

IMPORTANT: The editorial review process is now complete. PLOS will only permit corrections to spelling, formatting or significant scientific errors from this point onwards. Requests for major changes, or any which affect the scientific understanding of your work, will cause delays to the publication date of your manuscript.

If your institution or institutions have a press office, please notify them about your upcoming paper to help maximize its impact. If they'll be preparing press materials, please inform our press team as soon as possible -- no later than 48 hours after receiving the formal acceptance. Your manuscript will remain under strict press embargo until 2 pm Eastern Time on the date of publication. For more information, please contact globalpubhealth@plos.org.

Thank you again for supporting Open Access publishing; we are looking forward to publishing your work in PLOS Global Public Health.

Best regards,

Priyanka Roy, MD, MPH, PhD

Academic Editor

PLOS Global Public Health

***********************************************************

The reviewers are satisfied that you have adequately addressed their comments, and they are pleased with the resulting revisions to the manuscript.

Reviewer Comments (if any, and for reference):

Reviewer's Responses to Questions

**Comments to the Author**

1. If the authors have adequately addressed your comments raised in a previous round of review and you feel that this manuscript is now acceptable for publication, you may indicate that here to bypass the “Comments to the Author” section, enter your conflict of interest statement in the “Confidential to Editor” section, and submit your "Accept" recommendation.

Reviewer #2: All comments have been addressed

Reviewer #3: All comments have been addressed

**********

2. Does this manuscript meet PLOS Global Public Health’s publication criteria? Is the manuscript technically sound, and do the data support the conclusions? The manuscript must describe methodologically and ethically rigorous research with conclusions that are appropriately drawn based on the data presented.

Reviewer #2: Yes

Reviewer #3: Yes

**********

3. Has the statistical analysis been performed appropriately and rigorously?

Reviewer #2: Yes

Reviewer #3: Yes

**********

4. Have the authors made all data underlying the findings in their manuscript fully available (please refer to the Data Availability Statement at the start of the manuscript PDF file)?

The PLOS Data policy requires authors to make all data underlying the findings described in their manuscript fully available without restriction, with rare exception. The data should be provided as part of the manuscript or its supporting information, or deposited to a public repository. For example, in addition to summary statistics, the data points behind means, medians and variance measures should be available. If there are restrictions on publicly sharing data—e.g. participant privacy or use of data from a third party—those must be specified.

Reviewer #2: Yes

Reviewer #3: Yes

**********

5. Is the manuscript presented in an intelligible fashion and written in standard English?

PLOS Global Public Health does not copyedit accepted manuscripts, so the language in submitted articles must be clear, correct, and unambiguous. Any typographical or grammatical errors should be corrected at revision, so please note any specific errors here.

Reviewer #2: Yes

Reviewer #3: Yes

**********

6. Review Comments to the Author

Please use the space provided to explain your answers to the questions above. You may also include additional comments for the author, including concerns about dual publication, research ethics, or publication ethics. (Please upload your review as an attachment if it exceeds 20,000 characters)

Reviewer #2: Well done, no future comments.

Reviewer #3: I am satisfied with the authors' responses and the resulting changes to the paper.

**********

7. PLOS authors have the option to publish the peer review history of their article (what does this mean?). If published, this will include your full peer review and any attached files.

**Do you want your identity to be public for this peer review?**  If you choose “no”, your identity will remain anonymous but your review may still be made public.

For information about this choice, including consent withdrawal, please see our Privacy Policy.

Reviewer #2: **Yes:** Virak Sorn

Reviewer #3: No

**********
